# Optimal dose for the efficacy of asenapine in patients with schizophrenia: Real‐world data

**DOI:** 10.1002/npr2.12389

**Published:** 2023-11-05

**Authors:** Yoshiteru Takekita, Shuichi Hiraoka, Yasuhiro Iwama, Daisuke Matsui, Nobuatsu Aoki, Haruhiko Ogata, Toshiya Funatsuki, Toshiyuki Shimizu, Yuji Murase, Yosuke Koshikawa, Masaki Kato, Toshihiko Kinoshita

**Affiliations:** ^1^ Department of Neuropsychiatry Kansai Medical University Osaka Japan; ^2^ Medical Affairs Department Meiji Seika Pharma Co., Ltd. Tokyo Japan; ^3^ Regulatory & Datascience Department, Pharmaceutical Research & Development Division Meiji Seika Pharma Co., Ltd. Tokyo Japan; ^4^ Safety Vigilance & Management Department, Reliability & Quality Assurance Division Meiji Seika Pharma Co., Ltd. Tokyo Japan; ^5^ School of Psychiatry University of New South Wales Randwick New South Wales Australia; ^6^ Black Dog Institute Randwick New South Wales Australia

**Keywords:** asenapine, dose–response, neuropsychopharmacology, real‐world data, schizophrenia

## Abstract

**Aims:**

A meta‐analysis of short‐term studies revealed no significant differences between the doses of asenapine, 10 and 20 mg/day, in the acute treatment of schizophrenia. However, it should be noted that many patients from clinical practice were excluded, and the dose–response to asenapine in a real‐world setting is still unclear. Additionally, the dose–response in the maintenance phase is not clear. This study aimed to evaluate the differences in the efficacy of different asenapine doses in patients with maintenance phase of schizophrenia in a real‐world setting.

**Methods:**

This study conducted post‐marketing surveillance of asenapine in clinical settings in Japan. It followed patients diagnosed with schizophrenia who received asenapine for the first time for a maximum of 52 weeks. These patients were divided into two categories based on their average daily asenapine dosage: ≤10 mg/day and >10 mg/day. Asenapine efficacy was assessed by adjusting for patient demographics using multivariate logistic regression analysis, employing the Clinical Global Impression‐Global Improvement (CGI‐I) scale, which has seven categories.

**Results:**

A total of 2774 patients were included in the analysis. Of these, 1689 and 1085 patients were treated with asenapine ≤10 mg/day and >10 mg/day, respectively. The CGI‐I improvement rate was significantly higher in the asenapine >10 group (*p* = 0.012) after adjusting for patient background factors.

**Conclusion:**

These results suggest that asenapine doses >10 mg/day may be more effective than 10 mg/day in the treatment of schizophrenia; however, further studies are needed to confirm these findings.

## Abstract 2

A meta‐analysis of large short‐term studies found no significant difference between asenapine doses of 10 and 20 mg/day for acute schizophrenia treatment. However, these studies' homogeneous patient populations may not accurately represent the diversity seen in clinical practice. This study's new findings suggest that doses exceeding 10 mg/day may prove more effective for long‐term maintenance therapy in real‐world settings.

## Graphical abstract

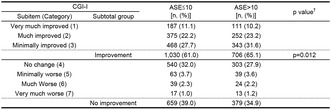

## 1 INTRODUCTION

Asenapine, a second‐generation antipsychotic, is well tolerated and significantly improves positive and negative symptom scale (PANSS) scores compared to placebo in randomized controlled trials (RCTs) of treatments for schizophrenia.^1^, ^2^ A meta‐analysis of short‐term acute phase studies revealed no significant difference between the improvement in the asenapine 10 mg/day and 20 mg/day groups, indicating that both doses were equally effective.^3^ However, in the relevant RCTs, patient characteristics were homogeneous across groups owing to randomization, and many patients in clinical practice, such as those with comorbidities and older people, were excluded. Moreover, a dose–response relationship with long‐term treatment in patients in the maintenance phase has not been previously reported. In this study, using data from the post‐marketing surveillance of asenapine in clinical settings,^4^ we examined the dose–response to asenapine in a real‐world setting, adjusting for patient background factors at the time of treatment initiation.

## 2 METHODS

Post‐marketing surveillance, which was approved by the Review Committee for Post‐marketing Surveillance of Meiji Seika Pharma Co., Ltd., was conducted in Japan between January 2017 and December 2019.^4^ Patients were diagnosed with schizophrenia based on operational diagnostic criteria conducted by a psychiatrist and receiving asenapine for the first time were followed up for a maximum of 52 weeks. The patient demographics, duration of asenapine administration, concomitant medications, adverse events, clinical laboratory values, and efficacy were investigated. All adverse events were coded according to the Medical Dictionary for Regulatory Activities/Japanese (MedDRA/J) version 23.0. Efficacy was assessed using the Clinical Global Impression‐Global Improvement (CGI‐I) scale [(1) very much improved, (2) much improved, (3) minimally improved, (4) no change, (5) minimally worse, (6) much worse, and (7) very much worse].

This study was conducted as post hoc analysis of a cohort study. Patients were divided into two groups receiving an average daily asenapine dose of ≤10 mg/day (ASE≤10) and >10 mg/day (ASE > 10), because the approved maintenance and maximum asenapine doses for schizophrenia in various countries are 10 and 20 mg/day, respectively.^3^ CGI‐I categories (1) to (2) were defined as effective, since CGI‐I category (3) corresponded to PANSS percentage change scores from 21 to 23,^5^ and the difference in the efficacy of asenapine between the two groups was assessed by adjusting for patient demographics using multivariate logistic regression analysis to identify the background factors that differed between the groups at a significance level of 0.1. Variables were selected using the stepwise method. Statistical analysis was also performed on background factors related to the continuation of asenapine. Ethical review of this study was approved by the institutional review board, and written informed consent was waived because of the retrospective design.

## 3 RESULTS

A total of 3425 patients from 543 centers were enrolled, and the questionnaires retrieved from 3364 patients were fixed. After excluding 571 patients who could not be evaluated using CGI‐I, 2794 patients were evaluated for efficacy. Of these, 1689 and 1085 patients were treated with ASE ≤ 10 and ASE > 10, respectively, and 20 patients received unknown doses. Therefore, 2774 patients were included in the analysis. The detailed patient characteristics are presented in Table 1.

**TABLE 1:** Patient's characteristics before the administration of asenapine.

		Total (*N* = 2774)	ASE ≤ 10 (*N* = 1689)	ASE > 10 (*N* = 1085)	*p* Value (χ^2^ test)
Male	[*n*, (%)]	1297 (46.8)	735 (43.5)	562 (51.8)	*p* < 0.001
Mean age	[years, mean (SD)]	46.9 (15.2)	47.3 (15.6)	46.4 (14.4)	ns^f^
Inpatient	[*n*, (%)]	1327 (47.8)	658 (39.0)	669 (61.7)	*p* < 0.001
Duration of illness^a^	[years, mean (SD)]	17.3 (14.3)	16.3 (14.4)	18.9 (14.0)	*p* < 0.001^f^
Body weight^b^	[kg, mean (SD)]	62.7 (15.3)	61.2 (14.9)	64.7 (15.6)	*p* < 0.001^f^
BMI^c^	[mean (SD)]	23.7 (5.0)	23.3 (5.0)	24.2 (5.1)	*p* < 0.001^f^
Hepatic dysfunction	[*n*, (%)]	133 (4.8)	63 (3.7)	70 (6.5)	*p* < 0.01
Renal dysfunction	[*n*, (%)]	39 (1.4)	17 (1.0)	22 (2.0)	ns
Diabetes	[*n*, (%)]	245 (8.8)	130 (7.7)	115 (10.6)	*p* < 0.05
Dyslipidemia	[*n*, (%)]	306 (11.0)	159 (9.4)	147 (13.5)	*p* < 0.01
Hypertension	[*n*, (%)]	273 (9.8)	134 (7.9)	139 (12.8)	*p* < 0.001
Extrapyramidal symptoms	[*n*, (%)]	376 (13.6)	191 (11.3)	185 (17.1)	p < 0.001
Anxiolytics/hypnotic sedatives	[*n*, (%)]	1261 (45.5)	704 (41.7)	557 (51.3)	p < 0.001
Anticonvulsant	[*n*, (%)]	631 (22.7)	336 (19.9)	295 (27.2)	*p* < 0.001
Antiparkinson agent	[*n*, (%)]	560 (20.2)	299 (17.7)	261 (24.1)	*p* < 0.001
Antipsychotics	[*n*, (%)]	2175 (78.4)	1266 (75.0)	909 (83.8)	*p* < 0.001
Number of antipsychotics^d^	[number of drugs, mean (SD)]	1.2 (1.0)	1.0 (0.9)	1.4 (1.1)	*p* < 0.001^f^
CP equivalent^e^	[mg/day, mean (SD)]	489.8 (502.1)	402.4 (451.5)	626.7 (545.3)	*p* < 0.001^f^
Treatment started with asenapine monotherapy	[*n*, (%)]	749 (27.0)	554 (32.8)	195 (18.0)	*p* < 0.001
Treatment phase					
Acute	[*n*, (%)]	1418 (51.1)	808 (47.8)	610 (56.2)	*p* < 0.001
Convalescent	[*n*, (%)]	277 (10.0)	178 (10.5)	99 (9.1)	
Stable	[*n*, (%)]	889 (32.0)	597 (35.3)	292 (26.9)	
Other	[*n*, (%)]	190 (6.8)	106 (6.3)	84 (7.7)	

Abbreviations: BMI, Body mass index; CP, Chlorpromazine; SD, standard deviation.

^a^ Total (*N* = 2187), ASE ≤ 10 (*N* = 1309) and ASE > 10 (*N* = 878).

^b^ Total (*N* = 1802), ASE ≤ 10 (*N* = 1008) and ASE > 10 (*N* = 794).

^c^ Total (*N* = 1694), ASE ≤ 10 (*N* = 934) and ASE > 10 (*N* = 760).

^d^ Total (*N* = 2760), ASE ≤ 10 (*N* = 1679) and ASE > 10 (*N* = 1081).

^e^ Total (*N* = 2738), ASE ≤ 10 (*N* = 1672) and ASE > 10 (*N* = 1066).

^f^ *t*‐test.

The efficacy was evaluated by defining the total percentage of CGI‐I categories (1), (2), and (3) as the improvement rate. When the independence of the improvement rates in ASE ≤ 10 and ASE > 10 was tested without adjusting for background factors using the χ^2^ test, a significant difference (*p* = 0.030) was observed. A logistic regression analysis with asenapine monotherapy/combination therapy and mean daily doses as factors was performed, and no significant interaction between them was identified (*p* = 0.0959). Therefore, multivariate logistic analysis using all patients was performed. The following four items were identified by multivariate logistic regression analysis: duration of illness, inpatient (vs. outpatient), anxiolytic/hypnotic sedatives (vs. without), and number of antipsychotics. The odds ratios (95% CI; *p*‐value) were 0.993 (0.986–1.000; *p* < 0.05), 1.390 (1.148–1.683; *p* > 0.001), 0.662 (0.552–0.794; *p* < 0.001) and 0.848 (0.771–0.933; *p* < 0.001), respectively (Table 2). Even after adjustment for multivariate logistic regression analysis, the ASE > 10 showed a significantly higher CGI‐I improvement rate than the ASE ≤ 10 (*p* = 0.012) (Table 3).

**TABLE 2:** Multivariate logistic regression analysis to determine the percentage of patients with improvement in Clinical Global Impressions‐global improvement (CGI‐I) scale.

Outcome: CGI‐I improvement	Odds ratio (95% CI)	*p* Value
>10 mg/day (vs. ≤10 mg/day)	1.276 (1.054–1.546)	*p* < 0.05
Duration of illness	0.993 (0.986–1.000)	*p* < 0.05
Inpatient (vs. outpatient)	1.390 (1.148–1.683)	*p* < 0.001
With anxiolytics/hypnotic sedatives (vs. without)	0.662 (0.552–0.794)	*p* < 0.001
Number of antipsychotics	0.848 (0.771–0.933)	*p* < 0.001

**TABLE 3:** Evaluation of Clinical Global Impressions‐global improvement (CGI‐I) based on average asenapine dose.

CGI‐I		ASE ≤ 10 [*n*, (%)]	ASE > 10 [*n*, (%)]	*p* Value^a^
Subitem (category)	Subtotal group			
Very much improved (1)		187 (11.1)	111 (10.2)	
Much improved (2)		375 (22.2)	252 (23.2)	
Minimally improved (3)		468 (27.7)	343 (31.6)	
	Improvement	1030 (61.0)	706 (65.1)	0.012
No change (4)		540 (32.0)	303 (27.9)	
Minimally worse (5)		63 (3.7)	39 (3.6)	
Much Worse (6)		39 (2.3)	24 (2.2)	
Very much worse (7)		17 (1.0)	13 (1.2)	
	No improvement	659 (39.0)	379 (34.9)	

^a^ Adjusted by multiple logistic regression analysis (details are shown in Table 2).

The mean days of treatment duration in both groups was 202.5 ± 156.3 in ASE ≤ 10 and 238.5 ± 137.4 in ASE > 10 (mean ± SD). In addition, the continuation rate was 45.6% in ASE ≤ 10 and 50.0% in ASE > 10, and the highest reasons for discontinuation were the occurrence of adverse events in ASE ≤ 10 and lack of efficacy in ASE > 10 (Table S1). Adverse events found at a rate of 1% or greater in both ASE ≤ 10 and ASE > 10 are shown in Table S2. The main adverse events were akathisia, hypoesthesia of oral, and somnolence. There were no marked differences in adverse events expressed between the two groups.

Demographics and clinical characteristics that were significant differences in those who continued asenapine are shown in Table S3. In ASE ≤ 10, they were inpatient/outpatient, duration of illness, and treatment phase. Similarly, in ASE > 10, there was the significant difference in inpatient/outpatient as well as ASE ≤ 10, however, the tendency which was different from ASE ≤ 10. Moreover, there was a significant difference in mean age only in ASE > 10.

## 4 DISCUSSION

The present data suggest the benefit of using asenapine doses >10 mg/day. Efficacy was evaluated by considering ‘minimally improved’ as the CGI‐I category (3) or higher as improvement. This is because it has been reported that the relationship between CGI‐I ratings and percentage change follows a linear trend, such that ‘minimally improved’ corresponds to PANSS percentage change scores from 21 to 23.^5^ Moreover, a 20% reduction of PANSS has been used as the criterion for response to antipsychotic treatment in many clinical trials.^6^, ^7^, ^8^

A meta‐analysis of short‐term studies in patients with acute‐phase schizophrenia revealed that the effective asenapine dose for 95% of the population was 14.97 mg/day.^3^ Furthermore, in a post hoc analysis of a 6‐week RCT in patients with an acute exacerbation of schizophrenia, a cluster analysis based on PANSS Marder factor scores of patients receiving asenapine suggested that some patients required a dose of 20 mg/day.^9^ These findings, based on short‐term results, are consistent with the findings of this survey after 1 year of maintenance treatment.

However, in real‐world settings, patient background characteristics before asenapine administration differ substantially between ASE ≤ 10 and ASE > 10. The four items used for multivariate logistic regression analysis appeared to be characteristic of a patient population with severe schizophrenia and high‐dose requirements (Table 2). Nevertheless, even after adjusting for these four items, our data indicated a statistically significant difference between ASE ≤ 10 and ASE > 10. Intergroup differences may be attributable to dose escalation in some patients in clinical practice when attending physicians deem a dose exceeding the maintenance dose necessary. Therefore, our results, which showed higher efficacy in those with ASE > 10, are consistent with the results of a previous meta‐analysis on 14.97 mg/day efficacious asenapine dosing.^3^ On the other hand, as the factors related to the continuation of asenapine treatment, the common factors were found between the two groups, while inpatient/outpatient showed a different trend, and mean age was found only in ASE > 10. These results suggest that various factors are intricately involved in the continuation of asenapine.

Present data suggest that the dose–response of asenapine in a long‐term real‐world setting is consistent with short‐term acute phase treatment.

## 5 LIMITATION

A limitation of this study is the inability to eliminate the effects of confounding factors, including concomitant medications. Furthermore, dose efficacy was assessed using CGI‐I, disease severity was not evaluated.

## 6 CONCLUSIONS

The present data suggest that asenapine doses >10 mg/day may be beneficial for patients with schizophrenia. Therefore, the treatment of schizophrenia with asenapine should be based on each individual case, the clinical judgment of the attending physician, and the patient's preference, and measures such as exceeding the maintenance dose should be implemented when necessary. Further large‐scale studies are needed to verify these findings.

## AUTHOR CONTRIBUTIONS

YT conceived and designed of the study. DM conducted data acquisition. YI developed the statistical analysis plan and conducted statistical analysis. NA, HO, TF, TS, YM, and YK contributed to the interpretation of the results. SH drafted the original manuscript. MK and TK supervised the conduct of this study. All authors reviewed the manuscript draft and revised it critically on intellectual content. All authors approved the final version of the manuscript to be published.

## FUNDING INFORMATION

This research received no specific grant from any funding agency in the public, commercial, or not‐for‐profit sectors.

## CONFLICT OF INTERESTS STATEMENT

YT has received grant funding from the Japan Society for the Promotion of Science and speaker's honoraria from Meiji‐Seika Pharma, Sumitomo Pharma, Janssen Pharmaceutical, Otsuka, Eisai, MSD K.K. Daiichi‐Sankyo, Pfizer, UCB Japan, Takeda Pharmaceutical, Novartis, and Ono Pharmaceutical and is an Editorial Board member of *Neuropsychopharmacology Reports* and a co‐author of this article. To minimize bias, they were excluded from all editorial decision‐making related to the acceptance of this article for publication. NA received speaker honoraria from Daiichi‐Sankyo, Eli Lilly, Meiji‐Seika Pharma, Janssen Pharmaceutical, Eisai, and Otsuka. TF has received grant funding from the Japan Society for the Promotion of Science and speaker's honoraria from Sumitomo Pharma, Otsuka, Meiji Seika Pharma, Janssen Pharmaceutical, Takeda Pharmaceutical, Lundbeck, Viatris Inc, Eisai. TS has received speaker's honoraria from Meiji‐Seika Pharma, Sumitomo Pharma, Janssen Pharmaceutical, and Mochida Pharmaceutical. YM received speaker honoraria from Sumitomo Pharma, Meiji‐Seika Pharma, Takeda Pharmaceutical, Lundbeck. MK has received grant funding from the Japan Society for the Promotion of Science, SENSHIN Medical Research Foundation, and Japan Research Foundation for Clinical Pharmacology, and speaker's honoraria from Sumitomo Pharma, Otsuka, Meiji‐Seika Pharma, Eli Lilly, MSD K.K., GlaxoSmithKline, Pfizer, Janssen Pharmaceutical, Shionogi, Mitsubishi Tanabe Pharma, Takeda Pharmaceutical, Lundbeck, and Ono Pharmaceutical. TK received speaker honoraria from Otsuka, Sumitomo Pharma, Meiji‐Seika Pharma, Janssen Pharmaceutical, Eisai, Daiichi‐Sankyo, Takeda Pharmaceutical, Lundbeck, and Ono Pharmaceutical. HO and YK declare no conflict of interest associated with this manuscript. SH, YI, and DM are full‐time employees of Meiji Seika Pharma Co. Ltd.

## ETHICS STATEMENT

Approval of the Research Protocol by an Institution Review Board: This study was approved by the institutional review board.

Informed Consent: Written informed consent was waived because of the retrospective design.

Registry and the Registration No. of the study/Trial: N/A.

Animal studies: N/A.

## PERMISSION TO REPRODUCE MATERIAL FROM OTHER SOURCES

There is no material from other sources in this manuscript and supporting information.

## Supporting information


Table S1.

Table S2.

Table S3.

## Data Availability

The data are not publicly available due to ethical restrictions. As the dataset for this study was collected through regulatory‐mandated post‐marketing surveillance, specific consent for individual data disclosure was not obtained. Consequently, in accordance with ethical guidelines, we are unable to provide public access to the individual patient data.

## APPENDIX A POTENTIAL REFEREES

### A.1

Dr. Taro Kishi, Department of Psychiatry, Fujita Health University School of Medicine, 1‐98 Dengakugakubo, Kutsukake‐cho, Toyoake, Aichi 470‐1192, Japan. Email: tarok@fujita-hu.ac.jp. Dr. Hiroyoshi Takeuchi, Department of Neuropsychiatry, Keio University School of Medicine, 35 Shinanomachi, Shinjuku‐ku, Tokyo 160‐8582, Japan. Email: hirotak@dk9.so-net.ne.jp. Dr. Itaru Miura, Department of Neuropsychiatry, Fukushima Medical University School of Medicine, Hikarigaoka, Fukushima‐shi, Fukushima 960‐1101, Japan, Email: itaru@fmu.ac.jp. Dr. Ken Inada, Department of Psychiatry, Kitasato University School of Medicine, 1‐15‐1 Kitazato, Minami‐ku, Sagamihara‐shi, Kanagawa 252‐0374, Japan, Email: inadaken@kitasato-u.ac.jp. Dr. Tetsu Tomita, Department of Neuropsychiatry, Graduate School of Medicine, Hirosaki University, 5 Zaifu‐cho, Hirosaki, Aomori 036‐8562, Japan, Email: ttomita@hirosaki-u.ac.jp.
